# Health Care Policy Implementation Trial of Primary Human Papillomavirus–Based Cervical Screening in Denmark

**DOI:** 10.1002/ijc.70502

**Published:** 2026-04-27

**Authors:** Jesper Bonde, Jeppe Bennekou Schroll, Birgitte Tønnes Pedersen, Elsebeth Lynge, Marianne Waldstrøm, Petra Hall Viborg, Anna Frandsen, Rikke Holst Andersen, Susanne Nielsen, Doris Schledermann, Bettina Kjær Kristensen, Berit Andersen, the National Danish Cervical Cancer Screening Steering Committee

**Affiliations:** ^1^ Molecular Pathology Laboratory, Pathology, Afs. 134, AHH‐Hvidovre Hospital, Copenhagen University Hospital Hvidovre Capital Region of Denmark Denmark; ^2^ Department of Clinical, Research Cochrane Denmark & Centre for Evidence‐Based Medicine Odense (CEBMO) University of Southern Denmark Odense Denmark; ^3^ Open Patient Data Explorative Network (OPEN), Odense University Hospital Odense Denmark; ^4^ Department of Gynecology & Obstetrics Herlev‐Gentofte Hospital Herlev Denmark; ^5^ Department of Public Health University of Copenhagen Copenhagen Denmark; ^6^ Department of Pathology Aarhus University Hospital Aarhus Denmark; ^7^ Department of Clinical Medicine Aarhus University Aarhus Denmark; ^8^ The Danish Healthcare Quality Institute Copenhagen Denmark; ^9^ Department of Pathology Aalborg University Hospital Ålborg Denmark; ^10^ Department of Pathology Region Hospital Randers Randers Denmark; ^11^ Department of Pathology, Region Zealand Næstved Denmark; ^12^ Department of Pathology Lille Baelt Hospital Vejle Denmark; ^13^ UNICCA—University Research Clinic for Cancer Screening, Department of Public Health Programs, Randers Regional Hospital Randers Denmark; ^14^ Department of Clinical Medicine, Health Aarhus University Aarhus Denmark

**Keywords:** cervical cancer screening, cytology, health care policy, HPV

## Abstract

On mandate from the Danish Health Authority, a nationwide health care policy trial (HCP) of primary HPV screening for women aged 30–59 was initiated in 2021. The aim was to inform on the performance of HPV screening in comparison to existing cytology practice. The HCP trial included all Danish women between 30 and 59 years of age undergoing cervical cancer screening in 2021 (*n* = 178,323). Women with odd birthdays were screened with HPV screening (*n* = 91,517), while women born on even dates were screened with cytology (*n* = 86,806). The follow‐up period was individually censored at 18 months from the screening date. Outcomes and intention‐to‐treat analysis are reported. Overall, 9.1% and 3.0% were HPV or cytology positive on the index sample. Referral to colposcopy upon index sample was 1.9% with HPV and 2.1% with cytology screening. Referral to retest after a positive HPV index sample result was 7.7 higher for HPV than cytology. Combining index and retest rounds, 6.3% women had colposcopy in the HPV arm (RR 1.28) versus 4,9% in the cytology arm. HPV screening detected both more disease ≥ CIN2 (RR 1.51) and ≥ CIN3 (RR1.35). The national‐scale health care policy implementation trial showed that HPV‐based cervical cancer screening increased detection of histologically defined disease compared to the previous policy of cytology‐based screening for women 30–59 years of age. The HCP demonstrated the real‐life effect of HPV‐based screening over cytology‐based screening in detection of disease, but also details what to be expected in terms of health care resource requirements.

## Abstract (highlights)

What's new?

Uncertainty has persisted over whether human papillomavirus (HPV) testing outperforms high‐quality cytology in real‐world cervical cancer screening programs. To address this, Denmark launched a nationwide trial in 2021, implementing an HPV‐based screening strategy using specialized algorithms integrated with molecular and cytology triage to optimize follow‐up recommendations for women ages 30‐59. Relative to cytology, HPV screening detected more cervical disease and better identified high‐risk women, though it increased repeat testing and cumulative referrals over time. The findings show that HPV‐based screening improves cervical disease detection over cytology, supporting Denmark's transition in screening approach while highlighting opportunities to refine resource use.

## Graphical abstract

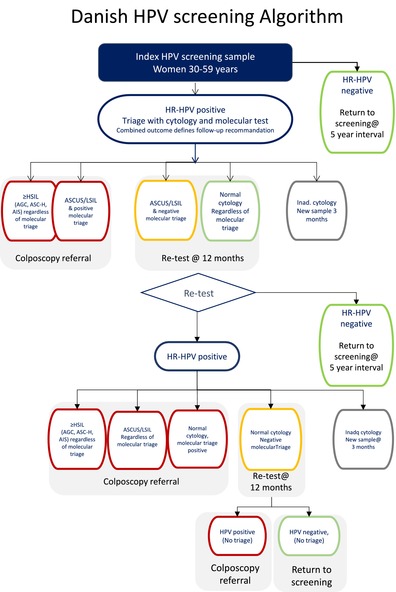

**Abbreviations**
CIN: cervical intraepithelial neoplasia
HCP: health care policy
HPV: human papillomavirus
ITT: intention to treat
LBC: liquid based cytology
LEEP: loop electrosurgical excision procedure
NSLS: National Steering Committee for cervical cancer screening
PP: per protocol
RR: Relative Risk

## 1 Introduction

Cervical cancer screening has been a health policy in Denmark for 50 years [1]. Globally, cervical cancer screening is recommended to reduce incidence of cervical cancer, and WHO has defined the goal of cervical cancer elimination as an incidence of ≤ 4 out of 100.000 women through a combination of vaccination, screening, and timely treatment [2, 3, 4, 5, 6, 7]. The European Union recommendations recommend human papillomavirus (HPV) based screening [8, 9], and randomized clinical trials, cross‐sectional studies and pilot implementation studies uniformly document that HPV screening provides superior cancer prevention over cytology [10, 11, 12, 13]. Use of HPV tests for screening is supported by international assay validation criteria to ensure optimal clinical sensitivity and specificity assay performance [14, 15, 16, 17].

First generation HPV screening algorithms largely relied on HPV positive findings to be triaged by cervical cytology [18, 19]. The second‐generation HPV algorithms utilized partial genotyping for HPV 16, HPV 18, and “other high risk” genotyping in combination with cytology triage [13, 19, 20, 21, 22, 23]. Countries that transitioned from cytology to HPV screening reported higher referral rates to colposcopy compared to previous cytology‐based programs [18, 20, 21, 24] calling for innovative approaches to increase the clinical specificity without compromising the superior clinical sensitivity of HPV screening.

In 2018 the Danish Health Authority mandated all five Danish Regions to initiate a health care policy trial (HCP) of primary HPV screening for women 30–59 years of age. An HCP trial is well suited where performance in real‐life settings needs evaluation before a definitive decision on change can be reached. As comparator to HPV‐based screening, the existing liquid‐based cytology (LBC) program was used [25, 26]. The Danish HCP trial combined HPV screening with three distinct triage options for HPV positive screening samples. Thus, LBC was a fixture in combination with either (1) p16ink4a/ki67 (CINtecplus), (2) partial genotyping for HPV 16 and HPV 18, other high‐risk HPV, or (3) extended genotyping reporting 6 individual genotypes and 8 genotypes reported in three separate pools.

The aim of the current study was to evaluate the acceptability and effectiveness of HPV screening in context of the Danish HCP trial for cell samples received during the first year of implementation (2021), with 18 month individual follow‐up time. We used index sample results and follow‐up outcomes to compare HPV‐based screening to cytology‐based screening. We report the intention‐to‐treat (ITT) outcome, that is, the total amount of histological abnormalities identified in the two groups regardless of whether the changes were found after a screening result that would lead to referral to gynecologist.

## 2 Methods

### 2.1 Setting

The Danish cervical cancer screening program is a free‐of‐charge public health program operated by the five Danish Regions. National guidelines are issued by the Danish Health Authority, and the National Steering Group for Cervical Cancer Screening (NSLS) is mandated to implement these guidelines in a coordinated way across regions. The cervical cancer screening program is exclusively operated by public hospitals.

Women aged 23–64 are personally invited for screening unless they have already a cell‐sample registered in the Danish Patobank within the recommended screening interval. In case of non‐participation, up to two reminders are sent 3 and 6 months after the initial invitation. Invitations and reminders are delivered by digital mail using the Danish cross‐governmental digital service infrastructure.

Women aged 23–49 are invited for screening every 3 years using cytology, whereas women 50–59 years are invited for screening every 5 years using cytology, and women 60–64 years are invited once to be screened using HPV DNA test as a program exit test [11, 25, 27, 28].

All cervical cancer screenings are conducted using clinician collected LBC. National quality criteria govern the eligibility of commercial HPV assays for use in the Danish screening program.

The study aimed to evaluate and compare the following outcomes between cytology (“control”) and HPV screening (“intervention”): (i) referral to colposcopy, (ii) referral to follow‐up testing (12 and 24 month in the HPV arm; 6 and 18 month in the cytology arm), (iii) number of procedures (cytologies, CINtec plus, colposcopies, histologies), (iv) CIN2+ and CIN3+ detection by screening arm. All results are reported as intention‐to‐treat (ITT).

### 2.2 Study Population and Screening Allocation

This study included all Danish women aged 30–59 years undergoing screening between 4th January and 31st December 2021.

**FIGURE 1:**
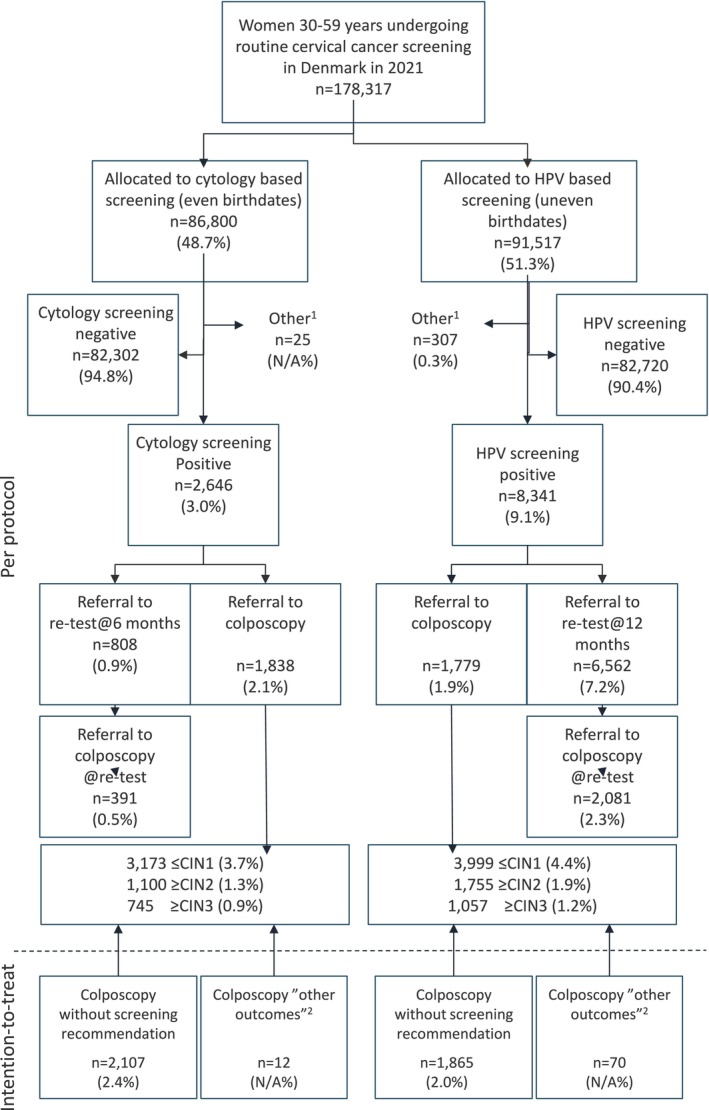
CONSORT style flow diagram showing the inclusions and exclusions made and observed in the health care policy implementation.

Samples from women with an odd birthday were screened with primary HPV screening (*n* = 91,517), while samples from women born on even dates were screened with primary cytology (*n* = 86,806). For practical reasons, allocation to cytology or HPV screening was done automatically by the national screening module upon reception of a sample. See Figure 1 for CONSORT style flow chart.

Invitations and reminders were used as per routine. Minor changes in the wording of the invitation letters included information related to HPV testing or cytology. As per routine, all women had to see a physician to have a sample taken. The follow‐up period for the present study ended individually 18 months from the date of index sample.

### 2.3 Exclusion Criteria

Samples from women with prior abnormalities were excluded. These were defined as first follow‐up after Loop electrosurgical excision procedure (LEEP) within 5 years, second follow‐up after LEEP if additional follow‐up was recommended, sample preceded by a test where additional follow‐up was recommended (within 1.5 years), preceded by previous histo‐pathology abnormalities (within 2 years), or preceded by any cervix cytology within 1.5 years prior. Remaining samples were screening samples.

At time of HCP implementation, one department in the Region of Southern Denmark was engaged in an HPV implementation trial since 2017 [29, 30]. All samples from this laboratory were excluded from the main analysis. Data from this department for the period 4th January 2021 through 31st December 2021 are reported solely in Tables S2 and S3 and serve as an indicator of future incidence HPV‐screening compared with the prevalence screening in the trial.

### 2.4 Sample Collection and Testing

All cervical cancer screening samples were collected in SurePath LBC medium (BD Diagnostics, Sparks, MD) using a combi brush, spatula or endocervical brush (Rovers, Oss, The Netherlands). Samples were exclusively collected by general practitioner clinics, gynecological specialist clinics or hospital departments. Sample collection was independent of subsequent allocation to either HPV or cytology modality.

Two of five regions used Roche Cobas 4800 HPV test, and one cobas 6800 (Roche Diagnostics, Pleasanton, CA) [16, 31], whereas two regions used the BD Onclarity HPV test (BD Diagnostics, Sparks, MD) [16, 32, 33, 34] (Table S1A–C). Each HPV test constituted approx. 50% of the total national HPV test activity. In this analysis, HPV test results are reported uniformly regardless of test platform.

In the HPV arm, HPV negative samples were returned to the next screening round. Invalid HPV samples were tested twice on the original sample, and if consistently invalid, a new sample was requested. For cytology, conclusively inadequate samples elicited referral for a new sample.

In the HPV arm, positive screening samples were referred for triage using three methods. One triage method was assigned per Region. Triage methods included (1) LBC and CINtecplus [35, 36] IHC (Roche, Rottkreutz, CH) (Regions Central Denmark and South Denmark) (2) LBC/HPV16, HPV 18, other HR (Regions Zealand and Northern Denmark), whereas triage (3) LBC and extended genotyping was assigned to the Capital Region of Denmark. Each triage method accounted for 43%, 22%, and 35% of the national screening volume, respectively [37].

**FIGURE 2:**
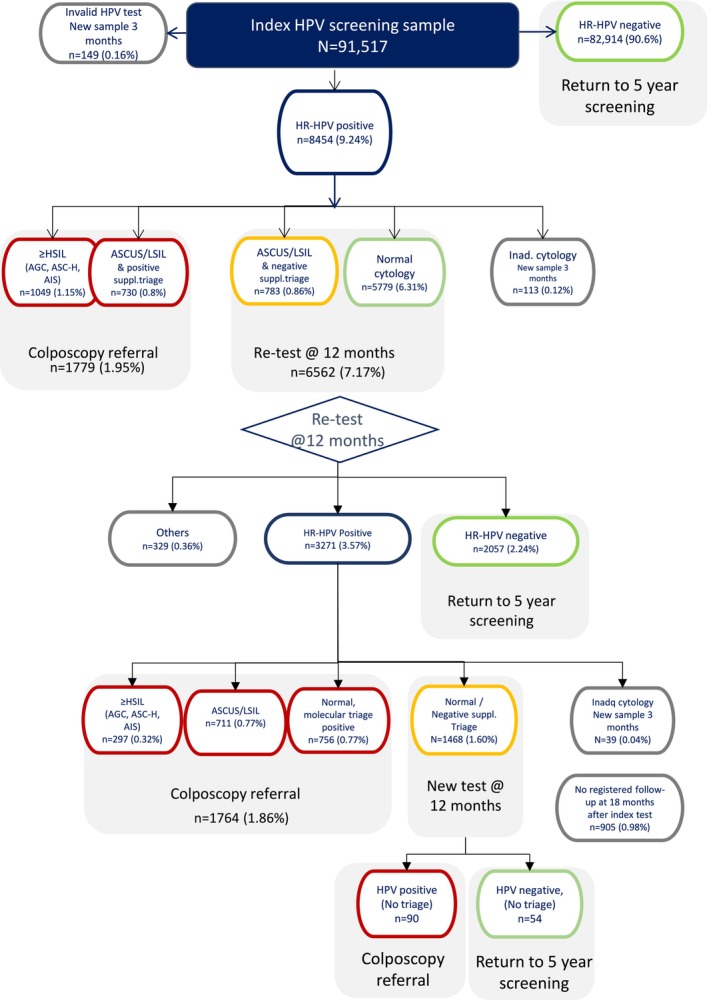
HPV algorithm with any HPV screened cervical sample result following the first registered sample in the study period. Percentages in () are out of all registered index samples.

All HPV positive screening tests were triaged by cytology, and outcomes reported using the Bethesda classification: negative for intraepithelial lesion or malignancy (normal), atypical squamous cells of undetermined significance (ASCUS), low‐grade squamous intraepithelial lesion (LSIL), high grade squamous intraepithelial lesion (HSIL), atypical glandular cells (AGC), typical squamous cells, cannot exclude HSIL (ASC‐H), adenocarcinoma in situ (AIS), and carcinoma. For analysis, HSIL, AGC, ASC‐H, AIS, and carcinoma are grouped as≥ HSIL. For HPV algorithm, see Figure 2.

Additional triage test results of (1) CINtecplus negative, (2) Other high risk, or (3) HPV35, 39, 45, 51, 56, 59, 66, or 68 were defined as “triage negative”, respectively, in combination with ASCUS, LSIL or normal cytology (See Figure 2). Triage outcomes of (1) CINtecplus positive, (2) HPV16 and/or HPV18, or (3) HPV16, 18, 31, 33/58, 52 were defined as “triage positive”. Management algorithms for each of the three triage methods can be found in Table S1A–C, however, a detailed reporting of the outcomes of the three triage methods is outside the scope of this analysis.

**FIGURE 3:**
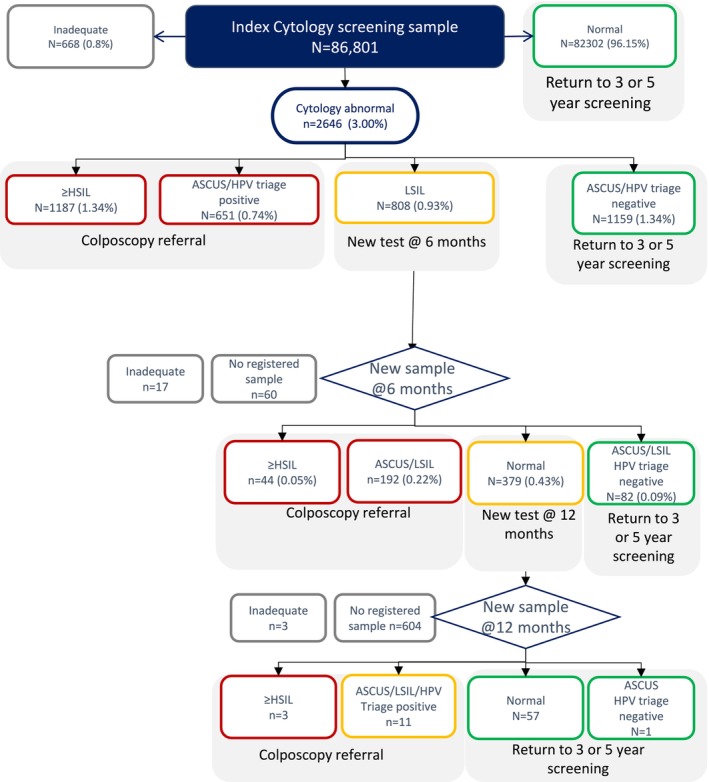
Cytology algorithm with any cytology screened cervical sample result following the first registered sample in the study period. Percentages in () are out of all registered index samples.

The cytology arm adhered to the 2012 Danish Cervical cancer screening guideline including triage of all ASCUS outcomes with HPV test before reporting. ASCUS with HPV negative triage outcomes is per national algorithm returned to next screening round. ASCUS/HPV positive in triage are referred to colposcopy. LSIL was only triaged with HPV test at 6 months re‐test as per national guideline (For cytology algorithm, see Figure 3).

In Denmark, all women examined by a gynecologist after a positive screening test have cervical biopsies and endocervical curettage/cell‐sampling regardless of colposcopy impression. Cervical biopsy histology outcomes are reported as negative for intraepithelial lesion or malignancy (normal), cervical intraepithelial neoplasia grade 1–3 (CIN1–CIN3), cervical intraepithelial neoplasia not otherwise specified (CIN NOS), AIS or carcinoma. For analysis, normal histology and CIN1 constitute ≤ CIN1, whereas CIN2, CIN NOS, CIN3, AIS, and carcinoma constitute ≥ CIN2. CIN3, AIS and carcinoma constitute ≥ CIN3.

All pathology departments conducting cervical cancer screening diagnostics report the result of the screening test using SNOMED codes to the Danish Pathology Data Bank, which contains all cytological and histological diagnoses reported in Denmark.

### 2.5 Data Sources and Analysis

All five regions initiated the HCP trial on the 4th of January 2021. The first registered screening sample during the recruitment period was defined as the index sample. Referral was defined as the action recommended based on the combined results of the index sample and the triage. The histology outcome of the screening was defined as the most severe histology diagnosis observed within 18 months of the index sample, regardless of when this histology was performed.

Register based follow‐up was conducted for all outcomes for each woman during the first 18 months following her index‐sample, with the last data retrieval undertaken in November 2023. All women who had a screening sample on record were included in the ITT analysis. Data were retrieved by the Danish Healthcare Quality Institute on behalf of NSLS.

For analysis in the present study, we restricted the triage outcomes to be cytology/molecular triage combined without further delineation of the individual triage strategies (Figure 2). Comprehensive reporting of the outcomes by individual triage strategy is outside the scope of this paper. For the entire ITT‐population, we reported on the number of women in the two arms by 5‐year age‐groups, on index‐sample result and recommended action based on the triage, and on the histopathology outcome. For women with a positive index sample, we reported on the triage result. Relative risk (RR) including 95% confidence intervals (CI) was used to compare proportions of women with a given outcome in the HPV‐arm compared to the cytology‐arm.

Statistical analysis was performed using SAS version 9.4 (SAS Institute Inc., Cary, NC, USA). The study is reported as per the CONSORT guideline.

## 3 Results

After exclusions, 178,317 women in the calendar year of 2021 had one sample defined as screening sample (index sample). No significant regional differences were observed in participation (data not shown). Of these, a total of 91,517 (51%) were allocated for HPV screening based on birthday and 86,800 (49%) for cytology screening (Table 1). During registration, 307 women, born on even dates, erroneously were screened with HPV whereas 25 women born on uneven dates erroneously were screened with cytology. As per ITT, the outcomes of these samples are counted within the allocated arm. The difference in number of women in each allocation arm reflects more uneven dates per calendar year. Age distribution between arms was similar (Table 1). Of all invited women, no differences in participation between cytology and HPV screening were observed, indicating equal acceptability (data not shown).

**TABLE 1:** Number of women (30–59 years) undergoing screening in Denmark, 4th January–31st December 2021 by allocation arm.

Age (years)	HPV screening		Cytology screening		Total
	*n*	% (95% CI)	*n*	% (95% CI)	*n*
All	91,517	51.3 (51.1–51.6)	86,800	48.7 (48.5–48.9)	178,317
30–34	17,007	18.6 (18.3–18.8)	16,177	18.6 (18.4–18.9)	33,184
35–39	16,271	17.8 (17.5–18.0)	15,516	17.9 (17.6–18.1)	31,787
40–44	17,004	18.6 (18.3–18.8)	16,187	18.7 (18.4–18.9)	33,191
45–49	18,923	20.7 (20.4–20.9)	17,829	20.5 (20.3–20.8)	36,752
50–54	11,645	12.7 (12.5–12.9)	10,905	12.6 (12.3–12.8)	22,550
55–59	10,667	11.7 (11.5–11.9)	10,186	11.7 (11.5–12.0)	20,853

*Note:* Primary HPV based screening versus Primary cytology screening (index samples 04.01.2021–31.12.2021, ITS‐population). Attender % is based on total population. Age group % is within arm.

In the HPV‐arm (Table 2), 8454 (9.1%, 95% CI 8.9–9.3) were found positive on the index sample, compared with 2646 (3.0%, CI 2.9–3.2) in the cytology‐arm (Table 2). Invalid HPV tests constituted 149 samples (0.2%, 95% CI 0.1–0.2) and 668 inadequate cytology samples (0.8%, 95% CI 0.7–0.8), respectively. In total, 6562 women referred to the 12 months retest in the HPV arm were (7.2%, 95% CI 7.0–7.3) (Figures 2 and 3, Table S1A–c), whereas cytology‐based referrals to the 6 months retest for LSIL findings was 808 women (0.9%, 95% CI 0.9–1.0). Referral to colposcopy after the index sample in the HPV arm was 1779 women (1.9%, 95% CI 1.9–2.0) compared with 1838 (2.1%, 95% CI 2.0–2.2) in the cytology‐arm (Table 2). Using the cytology index sample as reference, referral to retest after a positive HPV index sample had an RR of 7.70 (95% CI 7.16–8.28, Table 2). An age dependent variation was observed with RR 9.55 (95% CI 7.19–12.68) for women aged 55–59 and RR 6.16 (95% CI 5.27–7.20) for women aged 45–49 years. Referral to colposcopy after a positive index sample had an RR 0.92 (95% CI 0.86–0.98), varying from RR 1.03 (95% CI 0.88–1.20) amongst women 40–44 years to RR 0.67 (95% CI 0.54–0.82) amongst women aged 50–54.

**TABLE 2:** Index sample result and recommended action based on triage by allocated study arm—Primary HPV based screening versus Primary cytology screening (index samples 04.01.2021–31.12.2021, ITT‐population).

Screening outcome	Age (years)	HPV screening *n* = 91,517		Cytology screening *n* = 86,800		Total	
		*n*	% (95% CI)	*n*	% (95% CI)	*n*	RR (95% CI)
Index sample negative (return to screening)	All	82,720	90.4 (90.2–90.6)	82,302	94.8 (94.7–95.0)	165,022	0.95 (0.95–0.96)
	30–34	14,612	85.9 (85.4–86.4)	15,224	94.1 (93.7–94.5)	29,836	0.91 (0.91–0.92)
	35–39	14,482	89.0 (88.5–89.5)	14,718	94.9 (94.5–95.2)	29,200	0.94 (0.93–0.94)
	40–44	15,497	91.1 (90.7–91.6)	15,428	95.3 (95.0–95.6)	30,925	0.96 (0.95–0.96)
	45–49	17,396	91.9 (91.5–92.3)	16,953	95.1 (94.8–95.4)	34,349	0.97 (0.96–0.97)
	50–54	10,778	92.6 (92.1–93.0)	10,318	94.6 (94.2–95.0)	21,096	0.98 (0.97–0.98)
	55–59	9955	93.3 (92.8–93.8)	9661	94.8 (94.4–95.3)	19,616	0.98 (0.98–0.99)
Index sample positive	All	8341	9.1 (8.9–9.3)	2646	3.0 (2.9–3.2)	10,987	3.00 (2.86–3.12)
	30–34	2280	13.4 (12.9–13.9)	684	4.2 (3.9–4.5)	2964	3.17 (2.92–3.44)
	35–39	1692	10.4 (9.9–10.9)	526	3.4 (3.1–3.7)	2218	3.07 (2.79–3.37)
	40–44	1447	8.5 (8.1–8.9)	451	2.8 (2.5–3.1)	1898	3.05 (2.75–3.39)
	45–49	1457	7.7 (7.3–8.1)	484	2.7 (2.5–3.0)	1941	2.84 (2.56–3.14)
	50–54	809	6.9 (6.5–7.4)	276	2.5 (2.2–2.8)	1085	2.74 (2.40–3.14)
	55–59	656	6.1 (5.7–6.6)	225	2.2 (1.9–2.5)	881	2.78 (2.40–3.23)
Index sample positive (return to screening after negative triage)	All	Not an algorithm outcome		1159	1.3 (1.3–1.4)	N/A	N/A
Index sample invalid/inadequate (referral to new sample)	All	149	0.2 (0.1–0.2)	668	0.8 (0.7–0.8)	817	0.21 (0.18–0.25)
Other^a^	All	307	0.3 (0.3–0.4)	25	N/A	332	N/A
Total	All	91,517		86,800			
Referral to retest after positive index sample^a^	All	6562	7.2 (7.0–7.3)	808	0.9 (0.9–1.0)	7370	7.70 (7.16–8.28)
	30–34	1762	10.4 (9.9–10.8)	192	1.2 (1.0–1.4)	1954	8.73 (7.53–10.12)
	35–39	1320	8.1 (7.7–8.5)	160	1.0 (0.9–1.2)	1480	7.87 (6.69–9.26)
	40–44	1125	6.6 (6.2–7.0)	153	0.9 (0.8–1.1)	1278	7.00 (5.92–8.28)
	45–49	1171	6.2 (5.8–6.5)	179	1.0 (0.9–1.2)	1350	6.16 (5.27–7.20)
	50–54	664	5.7 (5.3–6.1)	72	0.7 (0.5–0.8)	736	8.64 (6.78–11.00)
	55–59	520	4.9 (4.5–5.3)	52	0.5 (0.4–0.7)	572	9.55 (7.19–12.68)
Referral to colposcopy after positive index sample^a^	All	1779	1.9 (1.9–2.0)	1838	2.1 (2.0–2.2)	3617	0.92 (0.86–0.98)
	30–34	518	3.0 (2.8–3.3)	492	3.0 (2.8–3.3)	1010	1.00 (0.89–1.13)
	35–39	372	2.3 (2.1–2.5)	366	2.4 (2.1–2.6)	738	0.97 (0.84–1.12)
	40–44	322	1.9 (1.7–2.1)	298	1.8 (1.6–2.1)	620	1.03 (0.88–1.20)
	45–49	286	1.5 (1.3–1.7)	305	1.7 (1.5–1.9)	591	0.88 (0.75–1.04)
	50–54	145	1.2 (1.1–1.5)	204	1.9 (1.6–2.1)	349	0.67 (0.54–0.82)
	55–59	136	1.3 (1.1–1.5)	173	1.7 (1.5–2.0)	309	0.75 (0.60–0.94)
Total	All	8341		2646		10,987	

*Note:* Screening outcome % is within arm and age group. Table values are rounded to one digit except for the RR values.

Abbreviation: RR, Relative Risk Ratio for primary HPV screening compared to primary cytology screening as reference group.

^a^ Includes misclassified samples according to screening allocation.

Amongst HPV positive index samples, 1049 women had triage outcomes of ≥HSIL (12.6%, 95% CI 11.9–13.3), 730 had ASCUS/LSIL/molecular triage positive (8.8%, 95% CI 8.2–9.4), and 783 were ASCUS/LSIL/triage negative (9.4%, 95% CI 8.8–10.0), respectively (Table S3). HPV screening positive with NILM cytology triage constituted 5779 women (69.3%, 95% CI 68.3–70.3) of the HPV positive index samples (Table S3). No significant differences were observed between age groups with respect to triage outcome after HPV positive index samples.

Overall, 6562 women in the HPV arm were recommended a 12 month retest. Of these, 905 women had no test, and 368 women had inadequate/other test results. Out of the remaining 5289 women, 3232 (61%) remained HPV positive (Table 3). Of these, 297 were referred to colposcopy after ≥HSIL triage (9.2%, 95% CI 8.2–10.2), 265 had ASCUS/LSIL/molecular triage positive outcome (8.2%, 95% CI 7.3–9.2), 446 ASCUS/LSIL/triage negative (13.8%, 95% CI 12.6–15.0), and 756 normal/triage positive (23.4%, 95% CI 21.9–24.9), respectively (Table 3). Also, 1468 women (45.4%, 95% CI 43.7–47.2) were normal/triage low, resulting in referral to an additional retest in 12 months (Table 3). Overall, 2057 women (39%) of the index HPV positive women cleared the infection between index and 12 months retest.

**TABLE 3:** Overall histology outcome by allocated study arm—Primary HPV based screening versus Primary cytology screening (index samples 04.01.2021–31.12.2021, ITT‐population, 18 month follow‐up).

	HPV screening *n* = 91,517		Cytology screening *n* = 86,800		Total	RR (95% CI)
	*n*	% (95% CI)	*n*	% (95% CI)	*n*	
Total	5754^a^	6.3 (6.1–6.4)	4273^a^	4.9 (4.8–5.1)	10,027	1.28 (1.23–1.33)
Colposcopy on index sample	1738	1.9 (1.8–2.0)	1763	2.0 (1.9–2.1)	3501	0.95 (0.86–1.01)
Colposcopy@6 months (cytology only)	N/A	N/A	391	0.5 (0.4–0.5)	391	N/A
Colposcopy@12 months (HPV only)	2081	2.3 (2.2–2.4)	N/A	N/A	2081	N/A
Colposcopy without screening recommendation	1865	2.0 (1.9–2.1)	2107	2.4 (2.3–2.5)	3972	0.84 (0.79–0.89)
Colposcopy in women with other outcomes	70		12			

Histology outcome	Age (Years)	*n*	% (95% CI)	*n*	% (95% CI)		
≤ CIN1	Total	3999	4.4 (4.2–4.5)	3173	3.7 (3.5–3.8)	7172	1.20 (1.14–1.25)
	30–34	816	4.8 (4.5–5.1)	571	3.5 (3.3–3.8)	1387	1.36 (1.22–1.51)
	35–39	709	4.4 (4.1–4.7)	538	3.4 (3.2–3.8)	1247	1.26 (1.13–1.40)
	40–44	721	4.2 (3.9–4.6)	608	3.8 (3.5–4.1)	1329	1.13 (1.02–1.25)
	45–49	840	4.4 (4.2–4.7)	721	4.0 (3.8–4.3)	1561	1.10 (1.00–1.21)
	50–54	529	4.5 (4.2–4.9)	408	3.7 (3.4–4.1)	937	1.21 (1.07–1.38)
	55–59	384	3.6 (3.3–4.0)	327	3.2 (2.9–3.6)	711	1.12 (0.97–1.30)
≥ CIN2	Total	1755	1.9 (1.8–2.0)	1100	1.3 (1.2–1.3)	2855	1.51 (1.40–1.63)
	30–34	544	3.2 (2.9–3.5)	332	2.1 (1.8–2.3)	876	1.56 (1.36–1.78)
	35–39	369	2.3 (2.0–2.5)	248	1.6 (1.4–1.8)	617	1.42 (1.21–1.66)
	40–44	322	1.9 (1.7–2.1)	191	1.2 (1.0–1.4)	513	1.60 (1.34–1.92)
	45–49	262	1.4 (1.2–1.6)	165	0.9 (0.8–1.1)	427	1.50 (1.23–1.82)
	50–54	153	1.3 (1.1–1.5)	91	0.8 (0.7–1.0)	244	1.57 (1.22–2.04)
	55–59	105	1.0 (0.8–1.2)	73	0.7 (0.6–0.8)	178	1.37 (1.02–1.85)
≥ CIN3	Total	1057	1.2 (1.1–1.2)	745	0.9 (0.8–0.9)	1802	1.35 (1.23–1.48)
	30–34	346	2.0 (1.8–2.3)	227	1.4 (1.2–1.6)	573	1.45 (1.23–1.71)
	35–39	224	1.4 (1.2–1.6)	170	1.1 (0.9–1.3)	394	1.26 (1.03–1.53)
	40–44	188	1.1 (1.0–1.3)	135	0.8 (0.7–1.0)	323	1.33 (1.06–1.65)
	45–49	144	0.8 (0.6–0.9)	107	0.6 (0.5–0.7)	251	1.27 (0.99–1.63)
	50–54	93	0.8 (0.7–1.0)	52	0.5 (0.4–0.6)	145	1.67 (1.19–2.35)
	55–59	62	0.6 (0.5–0.7)	54	0.5 (0.4–0.7)	116	1.10 (0.76–1.58)

*Note:* Histology outcome % is within arm and age group.

Abbreviation: RR, Risk Ratio for primary HPV screening compared to primary cytology screening as reference group.

^a^ Others: HPV arm: *n* = 70 (0.08%), Cytology arm *n* = 12 (0.01%), Total: *n* = 82. “Others” represents registered colposcopies with biopsies conducted on indications not related to the screening program.

At 12 months' retest, 1468 women in the HPV arm were recommended a new retest at 24 months. Of these 144 (9,8%) women had follow‐up at 18 months, and 37,5% cleared the infection (Figure 2).

**FIGURE 4:**
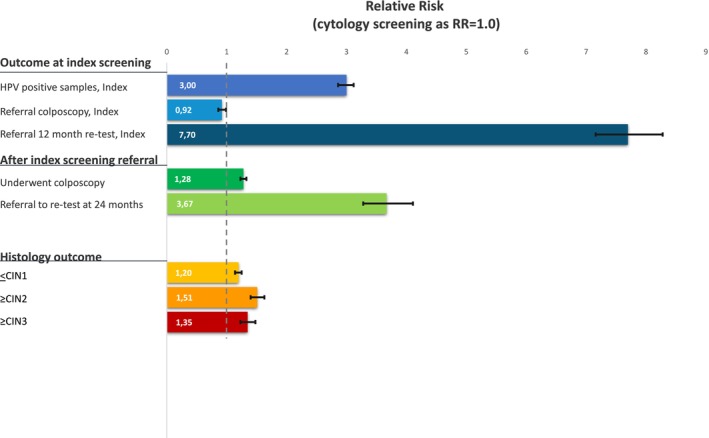
Summary of main trial results. Relative risk (RR) and 95% confidence interval (CI) for comparison between HPV arm and cytology arm in Health care policy trial of primary human papillomavirus–based cervical cancer screening in Denmark.

For cytology, of the 2646 cytology abnormal findings (3.3%), 1187 (31.2%, 95% CI 29.7–32.7) were ≥HSIL, 651 were ASCUS/HPV triage positive (17.1%, 95% CI 15.9–18.3), and 808 (21.1%, 95% CI 19.9–22.6) had LSIL (Table S4). Finally, 1159 (30.5%, 95% CI 29.0–32.0) were ASCUS, HPV triage negative (HPV, Table S4) and were returned to next screening round as per national guideline. The 808 women with LSIL were referred to retest at 6 months of which 60 had no test and 51 had inadequate/other results. Of the remaining 697 women (6.3%, 95% CI 4.6–8.4) showed ≥HSIL upon retest, 192 (27.5%, 95% CI 24.3–31.0) showed ASCUS or LSIL and HPV positive, 82 (11.8%, 95% CI 9.5–14.4) showed ASCUS or LSIL and HPV negative and returned to next screening round together with the 379 women (54.4%, 95% CI 50.6–58.1) with normal cytology being referred for a new sample in 12 months (Table S4, Figure 4).

In absolute numbers, 5754 women (6.3%, 95% CI 61.‐6.4) had colposcopy in the HPV arm versus 4273 women in the cytology arm (4.9%, 95% CI 4.8–5.1), RR 1.28 (95% CI 1.23–1.33) (Table 3). In the HPV‐arm, 1738 (1.9, 95% CI 1.8–2.0) women had colposcopy after referral based on a positive index sample, 2081 (2.3%, 95% CI 2.2–2.4) had colposcopy after a 12 month retest and a few after referral for 24 months retest. Overall, 1865 (2%, 95% CI 1.9–2.1) had colposcopy after a negative screening test. In the cytology‐arm 1763 had colposcopy after referral based on a positive index sample (2.0%, 95% CI 1.9–2.1), 391 (0.5%, 95% CI 0.4–0.5) after referral for 6 months retest and a few after 18 months retest. Overall, 2107 had colposcopy after a negative screening sample (2.4%, 95% CI 2.3–2.5). Colposcopy after a negative index sample was less common in the HPV‐arm than in the cytology‐arm, (RR 0.84, 95% CI 0.79–0.89). It should be noted that the number of women that will be recommended colposcopy through and entire screening round remains incomplete at this point. The numbers will increase slightly when all data are available for the 24 months follow‐up of women with positive HPV index sample, and for the 18 months follow‐up of women with positive cytology index sample.

Histology outcome showed that HPV screening generated more benign diagnoses than cytology with an RR of 1.20 (95% CI 1.14–1.25) for ≤ CIN1. This increase was less than the increase in detection of ≥ CIN2 in the HPV arm (RR 1.51, 95% CI 1.40–1.63) and for ≥ CIN3 at RR 1.35 (95% CI 1.23–1.48) (Table 3). The main trial results are summarized in Figure 4.

## 4 Discussion

A national health care professional (HCP) trial was initiated in 2021 to evaluate the benefits and limitations of HPV‐based screening compared with cytology‐based screening among women aged 30–59 years. HPV positivity was detected in 9.0% of participants, compared with 3.0% by cytology (RR = 3.0; Table 2). Overall, the rate of referral for retesting was nearly eightfold higher with HPV screening. This increase was primarily driven by the large number of women referred for 12 month retesting, particularly among younger age groups (Table 2). Most retest referrals (69%) were due to HPV‐positive screening samples with normal cytology and negative combined triage (Table S3). Notably, Swedish data indicate that HPV positivity with negative cytology is associated with elevated invasive cervical cancer risk [38], underscoring the need for appropriate follow‐up in this group.

Referrals to colposcopy after the index screening round were 1.9% in the HPV arm and 2.1% in the cytology arm. The slightly lower referral rate with HPV screening likely reflects the combined triage strategy, contrasting with other HPV implementation studies relying solely on cytology or partial genotyping. Including downstream retests over 18 months, 6.3% of women in the HPV arm and 4.9% in the cytology arm underwent colposcopy. The higher positivity rate in HPV screening resulted in significantly greater detection of ≥ CIN2 and ≥ CIN3 lesions (RR = 1.51; Table 3).

In the intention‐to‐treat (ITT) analysis, 28% more women received colposcopy following HPV screening than cytology over 18 months, consistent with Swedish data [38]. The index and 12 month retest each accounted for about half of all colposcopies (1779 and 1764, respectively; Figure 1 and Figure 2, Table S3). Among women persistently HPV‐positive at 12 months, 55% were referred for colposcopy, suggesting a possibly stringent triage algorithm. Disease yield among histologies was 31% (≥ CIN2) and 18% (≥ CIN3) in the HPV arm versus 26% and 17% in the cytology arm, respectively (Table 3). Detection of ≥ CIN2/≥ CIN3 was highest among younger women, largely driven by CIN2 (Table 3).

Overall, the Danish HCP trial findings align with results from the UK [18], Sweden [20], and a recent Cochrane review [24], confirming comparable relative sensitivity for HPV‐based detection of ≥ CIN2. Notably, a substantial proportion of colposcopies and biopsies occurred outside screening recommendations—comprising one‐third of all biopsies in the HPV arm and half in the cytology arm (Table 3)—suggesting clinician‐initiated activity beyond protocol. As the ITT analysis includes such activity, its impact on outcomes is under further investigation.

These findings indicate that while HPV screening increases overall diagnostic activity, particularly during the prevalence round, differences in disease yield compared with high‐quality cytology remain modest. With 18 months of follow‐up completed, only minimal additional dysplasia detection is anticipated at 24 months, unlikely to alter the main conclusions. How to triage HPV positive samples for best balance between sensitivity, specificity and cost remains a challenge. In this HCP, three different triage methods were used: HPV16/18 and cytology, Extended genotyping and cytology and HPV and CinTec. For outcomes of the triage method comparison, see Schroll et al. [39].

## 5 Strengths & Limitations

An HCP trial is particularly suited where performance in a real‐life setting needs evaluation before a definitive decision on change can be reached [20]. The allocation of women to the two screening modalities was per se not random, but by birth date, which was an operational choice. A strength of this HCP trial is that it represents a national implementation including all regions of Denmark. Only two HPV assays, BD Onclarity and Roche cobas (4800 and 6800), were used, which limits overall assay‐dependent HPV detection variability [40] between regions. Also, all cytology used for triage was conducted on BD SurePath.

A design limitation for performance comparison is that ASCUS positive cytology findings with concurrent triage HPV negative test are reported as normal. These women are returned to next recall for screening. Overall, ASCUS/HPV negative samples accounted for 1/3 of all cytology screening positive women (Table S4, Figure 3) but given the HPV negative status not eliciting a referral for follow‐up. This Danish cytology practice inherently favors cytology over HPV screening when evaluating on the number of referrals. This practice also reduces the difference between HPV and cytology screening in ≤ CIN1, ≥ CIN2, and ≥ CIN3 detection as it is reasonable to expect that if ASCUS/HPV negative samples had been referred for follow up, more colposcopies without detection of ≥ CIN2 would result in the cytology arm. In a longer perspective, the HCP trial consists by‐and‐large of unvaccinated women. A recent study conclusively shows that the positive predictive value of cytology will decline as childhood HPV vaccinated women enter the screening program [38]. This, along with the increased detection of disease by HPV screening over cytology, makes HPV screening the definitive choice.

This HCP trial presents the national prevalence round of HPV‐screening compared to long‐standing cytology screening and prevalence screening with HPV technology is well known to generate a higher rate of positives than the subsequent rounds. One department in Denmark has operated an implementation trial for HPV screening since 2017 [29] and is therefore not included in the national HCP trial. Acknowledging the differences in design between the national HCP trial and the local trial in this department, data retrieved for the same period from the local trial showed that 6.5% of the screened women were HPV positive at the 2nd screening round (incidence screening) compared with the 9% found in the HCP trial (Table S2). Also, the referral to colposcopy after the index‐sample in the incidence HPV screening was reduced to 1.3% versus the 1.9% found in this HCP trial (Tables S2 and S3). A similar difference between prevalence and incidence screening is well documented from other trials [19, 41], and it is reasonable to assume that the proportion of HPV positive women in subsequent rounds after implementation of HPV screening will be reduced.

Another limitation of the trial was the Danish Health Authority requested that the HCP trial was designed to ensure optimal patient safety. At the planning stage, in 2019, no data supported different intervals for retest for HPV screened positive women with concurrent normal cytology. It was therefore decided to refer all HPV positive women with normal cytology for retest at 12 months. Risk‐based screening strategies have developed since, that is, the Swedish HPV screening algorithm utilizing different retest intervals for HPV screening positive women with concurrent triage normal cytology dependent upon the HPV genotype detected and the woman's age [42, 43]. In our study, the major contribution to the retest after index HPV screening was HPV positive women with normal cytology (6.3% of the total of 7.2%, or 87.5%, Figure 2, Table S3). Whether the referral rate to retest in HPV screening will result in overdiagnosis is too early to determine, but another Swedish study found that HPV screening did not result in overdiagnosis over cytology after 13 years of follow‐up [44, 45]. Also, observational data from 500,000+ women in the UK screened with cytology or HPV found that the cumulative colposcopy rate was similar between screening methods over a screening round, but with the added benefit of HPV screening that high‐grade CIN was detected earlier [19]. Nevertheless, the increased number of screening positive women in the HPV‐arm merits a discussion on how the HPV algorithm can be adjusted to reduce overtreatment.

In this context, a derived observation of this analysis is the ability to assess viral persistence and clearance. Out of 6562 women referred for retest after HPV positive index sample, 5289 had a valid retest, and of those women 2057 were found HPV negative after 12 months. The overall viral clearance was thus 38.9% within the 12 months retest interval (Figure 2). Similarly, yet based on small numbers, an additional 37.5% of the women undergoing retest at 24 month had cleared any HPV infection detected at the 12 months re‐test. This leads to the following reflections: We note that 38.9% of the screened HPV positive women had cleared the infection at 12 months retest, and additionally 37,5% at 24 months. With the caveat that the 24 months retest data were limited, it nevertheless indicated that the current algorithm over‐manages women. In example, at 12 month retest some categories of HPV and triage outcomes could be allowed a longer interval before next test to allow for natural viral clearance. Also, single infection with HPV66 can lead to unnecessary referrals to retest. HPV66 had been down classified from the high‐risk HPV genotype group by the International Agency for Research on Cancer but remains a targeted genotype in most commercial HPV assays validated for screening including the two HPV screening tests used here [16].

## 6 Conclusions

The results of this large‐scale HCP trial have resulted in a recommendation from the National Health Authority to implement HPV screening for all women 30 years or older with 5 years interval for the whole group. The HCP trial demonstrated the real‐life effect of HPV‐based screening over cytology‐based screening in detection of disease, but also details what to be expected in terms of health care resource requirements because of an increased number of referrals and retests following the prevalence round. The HCP trial, however, does not assess the potential positive impact of the longer interval for HPV screening over cytology where intervals are extended from 3 to 5 years [7]. This possible benefit will manifest itself over time. The HCP trial also identified a significant activity not driven by screening‐related recommendations which requires further evaluation. Finally, the HCP trial already identified possible modifications of the HPV screening algorithm to reduce the immediate overtreatment and to ensure that clinical sensitivity and specificity are balanced as best possible.

## Author Contributions

**Jesper Bonde:** conceptualization, methodology, formal analysis, validation, investigation, resources, data curation, writing – original draft, writing – review and editing, visualization. **Jeppe Bennekou Schroll:** conceptualization, methodology, validation, formal analysis, investigation, data curation, writing – original draft, writing – review and editing, visualization. **Birgitte Tønnes Pedersen:** validation, formal analysis, software, data curation, visualization, writing – review and editing. **Elsebeth Lynge:** conceptualization, formal analysis, writing – original draft, writing – review and editing. **Marianne Waldstrøm:** conceptualization, formal analysis, investigation, resources, writing – original draft, writing – review and editing. **Petra Hall Viborg:** validation, software, data curation, writing – review and editing. **Anna Frandsen:** investigation, resources, writing – review and editing. **Rikke Holst Andersen:** investigation, writing – review and editing. **Susanne Nielsen:** investigation, writing – review and editing. **Doris Schledermann:** resources, writing – review and editing, investigation. **Bettina Kjær Kristensen:** conceptualization, formal analysis, writing – original draft, writing – review and editing. **Berit Andersen:** conceptualization, formal analysis, investigation, resources, writing – original draft, writing – review and editing.

## Funding

The HCP trial is a Danish Health Authority mandated health care initiative funded by the Danish Regions.

## Ethics Statement

The HCP trial is a Danish Health Authority mandated health care initiative in pursuance of the Danish Consolidation Act on Research Ethics Review of Health Research Projects. The HCP trial was not notifiable as per decision from the Medical Ethics Committee (R. No.: 1‐10‐72‐181‐20). No formal study protocol or informed consent was required.

## Conflicts of Interest

Jesper Bonde: Institution has received research funding and/or consumables at reduced price or for free to support research from BD Diagnostics (US), Biocartis (B), and Seegene (South Korea). Jesper Bonde has received honoraria for lectures from BD Diagnostics. Other authors declare no conflicts of interest.

## Supporting information


**Table S1A:** HPV screening with cytology and extended genotype triage.
**Table S1B:** HPV screening with combined cytology and CintecPlus triage.
**Table S1C:** HPV screening with combined HPV16/18 and cytology triage.
**Table S2:** HPV incidence screening 4.1.2021–31.12.2021 following clinical trial initiated 2017 using HPV screening with HPV16, 18, other‐HR algorithm.
**Table S3:** Triage result in women with positive index sample, and at 12 and 24 month retest—Primary HPV based screening (index samples 04.01.2021–31.12.2021, Positive index sample population).
**Table S4:** Triage result in women with positive index sample, and at 6 and 12 month retest—Primary cytology‐based screening (index samples 04.01.2021–31.12.2021, Positive index sample‐population).

## Data Availability

Further information is available from the corresponding author upon request.
