# Supplementary material for: Health Care Policy Implementation Trial of Primary Human Papillomavirus–Based Cervical Screening in Denmark
Source: Int J Cancer. 2026 Apr 27;159(6):1464–76. doi: 10.1002/ijc.70502 (PMC13397258; doi:10.1002/ijc.70502)
Supplement: Supplementary file 1 — Table S1A: HPV screening with cytology and extended genotype triage. Table S1B: HPV screening with combined cytology and CintecPlus triage. Table S1C: HPV screening with combined HPV16/18 and cytology triage. Table S2: HPV incidence screening 4.1.2021–31.12.2021 following clinical trial initiated 2017 using HPV screening with HPV16, 18, other‐HR algorithm. Table S3: Triage result in women with positive index sample, and at 12 and 24 month retest—Primary HPV based screening (index samples 04.01.2021–31.12.2021, Positive index sample population). Table S4: Triage result in women with positive index sample, and at 6 and 12 month retest—Primary cytology‐based screening (index samples 04.01.2021–31.12.2021, Positive index sample‐population). [file IJC-159-1464-s001.pdf]

Health care policy implementation trial of primary human papillomavirus–based cervical screening in Denmark

Jesper Bonde, Jeppe Bennekou Schroll, Birgitte Tønnes Pedersen, Elsebeth Lynge, Marianne Waldstrøm, Petra Hall Viborg, Anna Frandsen, Rikke Holst Andersen, Susanne Nielsen, Doris Schledermann, Bettina Kjær Kristensen, Berit Andersen on behalf of the National Danish Cervical Cancer Screening Steering Committee

Table of contents

Supplementary Table 1A HPV screening with cytology and extended genotype triage

Supplementary Table 1B HPV screening with combined cytology and Cintec®Plus triage

Supplementary Table 1C HPV screening with combined HPV16/18 and cytology triage

Supplementary Table 2 HPV incidence screening 4.1.2021-31.12.2021 following clinical trial initiated 2017 using HPV screening with HPV16, 18, other-HR algorithm

Supplementary Table 3. Triage result in women with positive index sample, and at 12- and 24-month retest - Primary HPV based screening (index samples 04.01.2021 - 31.12.2021, Positive index sample population)

Supplementary Table 4. Triage result in women with positive index sample, and at 6 and 12-month retest - Primary cytology-based screening (index samples 04.01.2021 - 31.12.2021, Positive index sample-population)

Supplementary Table 1A HPV screening with cytology and extended genotype triage

The Capital Region of Denmark<br>Number of women screened 4.1.2021-31.12.2021: 36,287<br>Proportion of all screened women: 40%<br>HPV positivity: 9%<br>HPV test: BD Onclarity HPV test™<br>Molecular triage: Extended genotyping included in HPV assay		
**Combined triage algorithm**<br>**Referral at index sample**		
**Molecular triage result**	**Cytology triage result**	**Referral Recommendation**
Independent of genotype	≥HSIL (incl. HSIL, AGC, ASC-H, AIS, carcinoma)	Colposcopy
Any of HPV16, 18, 31, 33, 52	ASCUS or LSIL	Colposcopy
Any of HPV35, 39, 45, 51, 56, (58), 66, 68	ASCUS or LSIL	New test in 12 months
Independent of genotype	NORMAL	New test in 12 months
HPV negative	Not performed	Return to screening
Independent of genotype	Inadequate	New test within 3 months
**Referral at new test @12 months**		
Independent of genotype	≥HSIL (incl. HSIL, AGC, ASC-H, AIS, carcinoma)	Colposcopy
Independent of genotype	ASCUS or LSIL	Colposcopy
Any of HPV16, 18, 31, 33, 52	Normal	Colposcopy
Any of HPV35, 39, 45, 51, 56, (58), 66, 68	Normal	New test in 12 months
HPV negative	Normal	Return to screening
Independent of genotype	Inadequate	New test within 3 months
**Referral at new test @ 12 months (24 months after index sample)**		

HPV positive, any genotype	Independent of cytology	Colposcopy
HPV negative	Independent of cytology	Return to screening

Supplementary Table 1B HPV screening with combined cytology and Cintec®Plus triage

The Central Denmark Region & Region of Southern Denmark<br>Number of women screened 4.1.2021-31.12.2021: 31,912<br>Proportion of all screened women: 35%<br>HPV positivity: 9.8% (Central Jutland), 10.6% (Southern Jutland)<br>HPV test: Cobas 4800 HPV test™<br>Molecular triage: CinTec Plus™ (Immunohistochemistry, IHC)		
**Combined triage algorithm<br>Referral at index sample**		
**Molecular triage result**	**Cytology triage result**	**Referral Recommendation**
Independent of IHC	≥HSIL (incl. HSIL, AGC, ASC-H, AIS, carcinoma)	Colposcopy
CINtec®plus positive	ASCUS or LSIL	Colposcopy
CINtec®plus negative	ASCUS or LSIL	New test in 12 months
Independent of IHC	Normal	New test in 12 months
HPV negative	Not performed	Return to screening
Independent of IHC	Inadequate	New test within 3 months
**Referral at new test @12 months**		
Independent of IHC	≥HSIL (incl. HSIL, AGC, ASC-H, AIS, carcinoma)	Colposcopy
Independent of IHC	ASCUS or LSIL	Colposcopy
CINtec®plus positive	Normal	Colposcopy
CINtec®plus negative	Normal	New test in 12 months
HPV negative	Normal	Return to screening

Independent of IHC	Inadequate	New test within 3 months
**Referral at new test @ 12 months (24 months after index sample)**		
HPV positive, any genotype	Independent of cytology	Colposcopy
HPV negative	Independent of cytology	Return to screening

Supplementary Table 1C HPV screening with combined HPV16/18 and cytology triage

The North Denmark Region & Region Zealand<br>Number of women screened 4.1.2021-31.12.2021: 23,309<br>Proportion of all screened women: 25%<br>HPV positivity: 9% (Zealand), 8.1% (Northern Jutland)<br>HPV test: Cobas 6800 HPV test™ (Zealand), BD Onclarity HPV test (Northern Jutland)<br>Molecular triage: HPV 16, HPV18 and other HR (31, 33, 35, 39, 45, 51, 52, 56, 58, 59, 66, 68)		
**Combined triage algorithm**<br>**Referral at index sample**		
**Molecular triage result**	**Cytology triage result**	**Referral Recommendation**
Independent of genotype	≥HSIL (incl. HSIL, AGC, ASC-H, AIS, carcinoma)	Colposcopy
Any of HPV 16 and HPV 18	ASCUS or LSIL	Colposcopy
other HR (31, 33, 35, 39, 45, 51, 52, 56, 58, 59, 66, 68)	ASCUS or LSIL	New test in 12 months
Independent of genotype	Normal	New test in 12 months
HPV negative	Not performed	Return to screening
Independent of genotype	Inadequate	New test within 3 months
**Referral at new test @12 months**		
Independent of genotype	≥HSIL (incl. HSIL, AGC, ASC-H, AIS, carcinoma)	Colposcopy
Independent of genotype	ASCUS or LSIL	Colposcopy

Any of HPV 16 and HPV 18	Normal	Colposcopy
other HR (31, 33, 35, 39, 45, 51, 52, 56, 58, 59, 66, 68)	Normal	New test in 12 months
HPV negative	Normal	Return to screening
Independent of genotype	Inadequate	New test within 3 months
**Referral at new test @ 12 months (24 months after index sample)**		
HPV positive, any genotype	Independent of cytology	Colposcopy
HPV negative	Independent of cytology	Return to screening

Supplementary Table 2 HPV incidence screening 4.1.2021-31.12.2021 following clinical trial initiated 2017 using HPV screening with HPV16, 18, other HR algorithm(26)

	Age (years)	n	% (95% CI)
All women screened 4.1.2021-31.12.2021	All	10804	
	30-34	1743	
	35-39	1817	
	40-44	2063	
	45-49	2443	
	50-54	1431	
	55-59	1307	
	Recommendation after screening sample		
Return to screening	All	10041	92.6 (92.1-93.1)
	30-34	1547	88.8 (87.2-90.2)
	35-39	1675	92.2 (90.9-93.4)
	40-44	1936	93.8 (92.7-94.8)
	45-49	2314	94.7 (93.8-95.6)
	50-54	1349	94.3 (92.9-95.4)
	55-59	1220	93.3 (91.9-94.6)
Index sample positive	All	707	6.5 (6.1-7.1)
	30-34	180	10.3 (8.9-11.9)
	35-39	131	7.2 (6.1-8.5)
	40-44	117	5.7 (4.7-6.8)
	45-49	120	4.9 (4.1-5.8)
	50-54	79	5.5 (4.4-6.8)
	55-59	80	6.1 (4.9-7.6)
Return to screening after negative triage		0	-
Referral to retest after positive index sample*	All	515	4.8 (4.4-5.2)
	30-34	128	7.3 (6.2-8.7)
	35-39	86	4.7 (3.8-5.8)
	40-44	83	4.0 (3.2-5.0)
	45-49	91	3.7 (3.0-4.6)
	50-54	61	4.3 (3.3-5.4)
	55-59	66	5.0 (3.9-6.4)
Referral to histology after positive index sample*	All	144	1.3 (1.1-1.6)
	30-34	41	2.4 (1.7-3.2)
	35-39	37	2.0 (1.4-2.8)
	40-44	25	1.2 (0.8-1.8)
	45-49	18	0.7 (0.4-1.2)
	50-54	13	0.9 (0.5-1.5)

	**55-59**	**10**	**0.8 (0.4-1.4)**
**Referral to new sample after invalid/inadequate sample***		**54**	**0.5 (0.4-0.6)**
**Other****		**50**	**0.5 (0.3-0.6)**

Supplementary Table 3. Triage result in women with positive index sample, and at 12- and 24-month retest - Primary HPV based screening (index samples 04.01.2021 - 31.12.2021, Positive index sample population)

HPV screening, positive index sample (N = 8341)									
**Triage**	≥HSIL*		ASCUS/LSIL and molecular triage positive		ASCUS/LSIL and molecular triage negative		normal cytology independent of molecular triage		
**Referral recommendation**	**Histology**		**Histology**		**Retest in 12 months**		**Retest in 12 months**		**Total (=100 %)**
**Age (years)**	**n**	**% (95% CI)**	**n**	**% (95% CI)**	**n**	**% (95% CI)**	**n**	**% (95% CI)**	**n**
All[1]	1049	12.6 (11.9-13.3)	730	8.8 (8.2-9.4)	783	9.4 (8.8-10.0)	5779[5]	69.3 (68.3-70.3)	8341
30-34	326	14.3 (12.9-15.8)	192	8.4 (7.3-9.6)	212	9.3 (8.1-10.6)	1550	68.0 (66.0-69.9)	2280
35-39	224	13.2 (11.7-14.9)	148	8.7 (7.4-10.2)	143	8.5 (7.1-9.9)	1177	69.6 (67.3-71.7)	1692
40-44	174	12.0 (10.4-13.8)	148	10.2 (8.7-11.9)	146	10.1 (8.6-11.8)	979	67.7 (65.2-70.1)	1447
45-49	148	10.2 (8.7-11.8)	138	9.5 (8.0-11.1)	145	10.0 (8.5-11.6)	1026	70.4 (68.0-72.8)	1457

50-54	86	10.6 (8.6-13.0)	59	7.3 (5.6-9.3)	90	11.1 (9.0-13.5)	574		71.0 (67.82-74.08)		809
55-59	91	13.9 (11.3-16.8)	45	6.9 (5.0-9.1)	47	7.2 (5.3-9.4)	473		72.1 (68.5-75.5)		656
**HPV arm @12 months retest, positive retest sample (N = 3232)** **(Data retrieval after 18 months)**											
**Triage**	≥HSIL*		ASCUS/LSIL and molecular triage positive		ASCUS/LSIL and molecular triage negative		NORMAL cytology/molecular triage positive		NORMAL cytology/molecular triage negative		**Total (=100 %)**
**Referral recommendation**	**Histology**		**Histology**		**Histology**		**Histology**		**Retest in 12 months**		
**Age (years)**	**n**	**% (95% CI)**	**n**	**% (95% CI)**	**n**	**% (95% CI)**	**n**	**% (95% CI)**	**n**		
All ages[2]	297	9.2 (8.2-10.2)	265	8.2 (7.3-9.2)	446	13.8 (12.6-15.0)	756	23.4 (21.9-24.9)	1468	45.4 (43.7-47.2)	3232
**HPV arm @24 months retest, positive re-retest sample (N = 144)** **(Data retrieval after 18 months)**[4]											

Triage	HPV positive	HPV negative	Total (=100%)	
**Referral recommendation**	**Histology**	**Return to screening**		

Age (years)	n	% (95% CI)	n	% (95% CI)		
All ages[3]	90	62.5 (54.1-70.4)	54	37.5 (29.6-45.9)	144	

Triage outcome % is within arm and age group
* HSIL, AGC, ASC-H, AIS, Carcinoma in situ, Carcinoma
1) invalid/inadequate: 149 samples
2)Inadequate/invalid (n=39), others incl. erroneously registered sample (n=239), no sample registered at 18 months follow-up (n=905)
3) others incl. erroneously registered sample (n=24), no sample registered at 18 months follow-up (n=1300)
4) data retrieval at 18 months after 31/12 2021

5) The difference from index sample to 12 months retest consist of a) HPV negative upon retest, b) women with a registered follow-up sample

Supplementary Table 4. Triage result in women with positive index sample, and at 6 and 12-month retest - Primary cytology-based screening (index samples 04.01.2021 - 31.12.2021, Positive index sample-population)

Cytology screening, Positive index sample (N=3805)									
Triage	≥HSIL*		ASCUS/HPV positive		LSIL		ASCUS/ HPV Negative		
Referral recommen-dation	Histology		Histology		Retest in 6 months		Return to screening		Total (=100%)
Age (years)	n	% (95% CI)	n	% (95% CI)	n	% (95% CI)	n	% (95% CI)	n
All	1187	31.2 (29.7-32.7)	651	17.1 (15.9-18.3)	808	21.2 (19.9-22.6)	1159	30.5 (29.0-32.0)	3805
30-34	323	38.4 (35.1-41.7)	169	20.1 (17.4-22.9)	192	26.6 (23.4-30.0)	147	21.9 (18.9-25.1)	842
35-39	250	34.7 (31.2-38.3)	116	16.1 (13.5-19.0)	160	30.4 (26.5-34.5)	158	37.1 (32.9-41.4)	721
40-44	190	26.1 (22.9-29.4)	108	14.8 (12.3-17.6)	153	21.0 (18.1-24.1)	195	38.1 (34.6-41.8)	729
45-49	179	26.2 (22.9-29.7)	126	18.4 (15.6-21.6)	179	26.2 (22.9-29.7)	278	29.1 (25.8-32.7)	683
50-54	129	28.2 (24.1-32.5)	75	16.4 (13.1-20.1)	72	15.7 (12.5-19.4)	199	39.7 (35.2-44.4)	458
55-59	116	31.2 (26.5-36.2)	57	15.3 (11.8-19.4)	52	14.0 (10.6-17.9)	182	39.5 (34.5-44.7)	372
Cytology arm, retest @ 6 months, Positive retest samples (N = 697) (Data retrieval after 18 months)									
	≥HSIL[1]		ASCUS/LSIL/HPV positive		NORMAL		ASCUS/LSIL/ HPV negative		
	Histology		Histology		Retest in 12 months		Return to screening		Total (=100%)
	n	% (95% CI)	n	% (95% CI)	n	% (95% CI)	N	% (95% CI)	n
All age groups	44	6.3 (4.6-8.4)	192	27.5 (24.3-31.0)	379	54.4 (50.6-58.1)	82	11.8 (9.5-14.4)	697
Cytology arm, retest @ 18 months, Positive re-retest samples (N = 72) (Data retrieval after 18 months)									
	≥HSIL*		ASCUS/LSIL/HPV positive		NORMAL		ASCUS/LSIL/ HPV negative		
	Histology		Histology		Return to screening		Return to screening		Total (=100%)
	n	% (95% CI)	n	% (95% CI)	n	% (95% CI)	n	% (95% CI)	n
All age groups	3	4.3 (0.9-11.7)	12	16.7 (8.9-27.3)	57	79.2 (68.0-87.8)	0	-	72

Triage outcome % is within arm and age group

1) HSIL, AGC, ASC-H, AIS, Carcinoma in situ, Carcinoma
2) data retrieval at 18 months after 31/12 2021
